# Supplementary material for: Anchor extension: a structure-guided approach to design cyclic peptides targeting enzyme active sites
Source: Nat Commun. 2021 Jun 7;12:3384. doi: 10.1038/s41467-021-23609-8 (PMC8185074; doi:10.1038/s41467-021-23609-8)
Supplement: Supplementary file 1 — Supporting information file [file 41467_2021_23609_MOESM1_ESM.pdf]

Supplementary Information

Anchor extension: a structure-guided approach to design cyclic peptides targeting enzyme active sites

Parisa Hosseinzadeh[1,&], Paris R. Watson[2,†], Timothy W. Craven[1,†], Xinting Li[1,†], Stephen Rettie[1,3,†], Fátima Pardo-Avila[4], Asim K. Bera[1], Vikram Khipple Mulligan[1,$], Peilong Lu[1,#], Alexander S. Ford[1], Brian D. Weitzner[1,¥], Lance J. Stewart[1], Adam P. Moyer[1,5], Maddalena Di Piazza[1], Joshua G. Whalen[1], Per Jr. Greisen[1,‡], David W. Christianson[2], David Baker[1,*]

1. University of Washington, Department of Biochemistry, Institute for Protein Design, Seattle, WA 98195, USA
2. Roy and Diana Vagelos Laboratories, Department of Chemistry, University of Pennsylvania, Philadelphia, PA 19104-6323 USA
3. Molecular and Cellular Biology Ph.D. Program, University of Washington, Seattle, WA 98195, USA
4. Department of Structural Biology, Stanford University School of Medicine, Stanford, CA 94305, USA
5. Molecular Engineering Ph.D. Program, University of Washington, Seattle, WA 98195, USA

† These authors contributed equally to this manuscript.
* Correspondence are directed to dabaker@uw.edu

Supplementary Figures

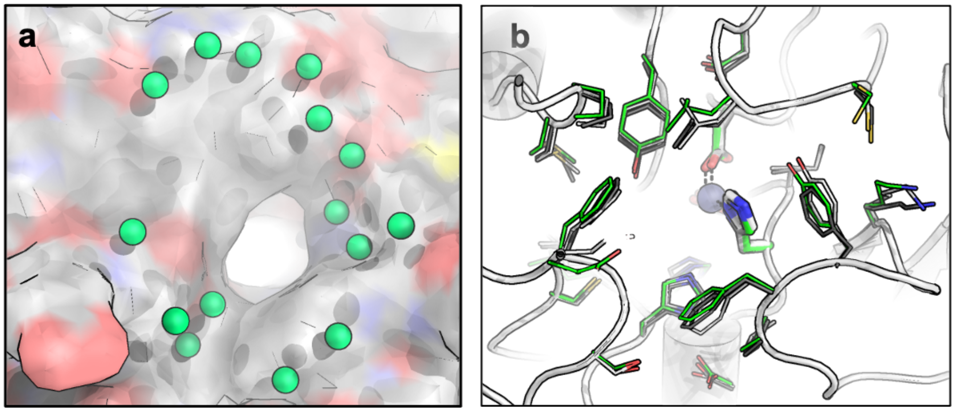

Supplementary Figure 1. Structural analysis of HDAC2 points to the challenges for designing selective and high affinity HDAC2 inhibitors. (a) Surface representation of HDAC2 binding pocket shows multiple polar atoms (pink = O, blue = N, yellow= S), as well as water molecules (green spheres). (b) Overlay of crystal structures of HDACs 1,2,3 (PDB IDs: 5ICN, 5IWG, 4A69 respectively) shows near identical structure and sequence at the binding pocket.

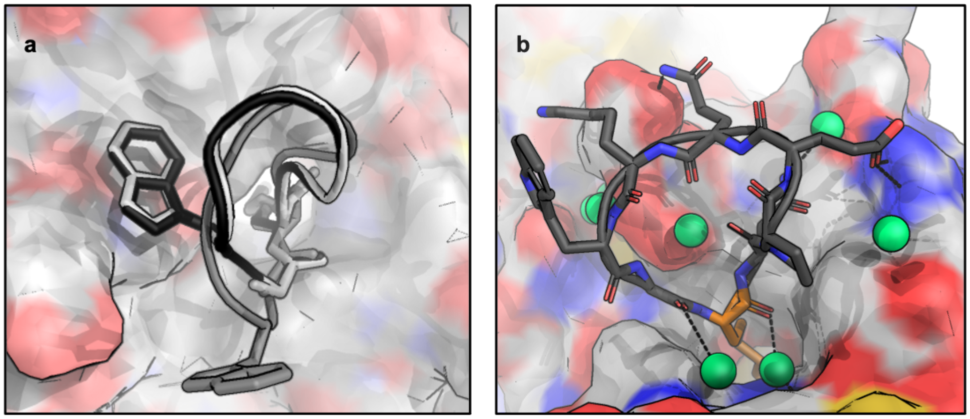

Supplementary Figure 2. Crystal structure of des1.1.0 shows two binding orientations. (a) Overlay of three chains in asymmetric crystallographic unit of des1.1.0 shows two distinct conformations. (b) des1.1.1 (Lys→Glu) stabilizes the least populated binding mode in all three chains, as designed.

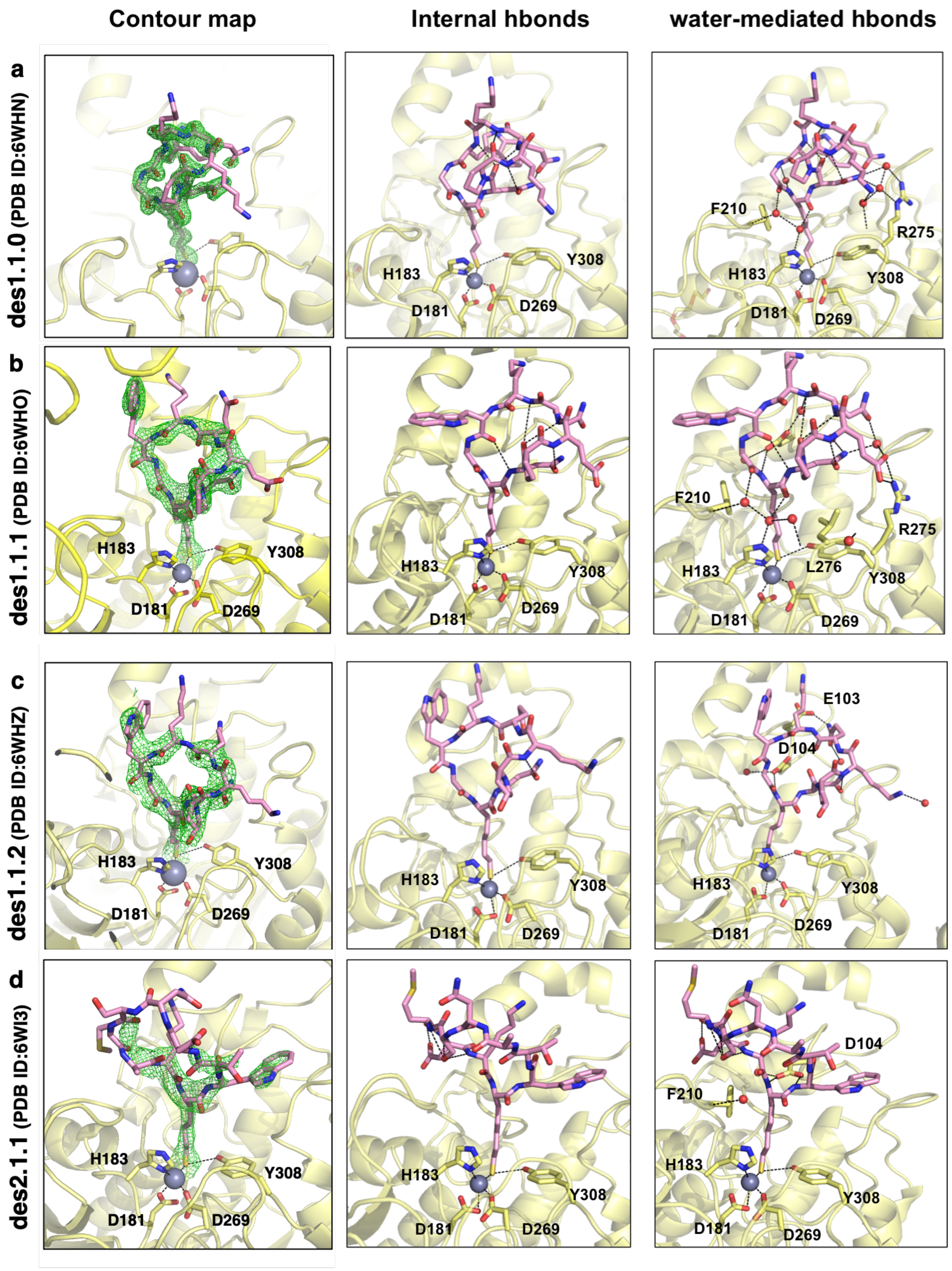

Contour map
Internal hbonds
water-mediated hbonds
a
des1.1.0 (PDB ID:6WHN)
H183
D181
D269
Y308
F210
R275
b
des1.1.1 (PDB ID:6WHO)
H183
D181
D269
Y308
F210
R275
L276
c
des1.1.2 (PDB ID:6WHZ)
H183
D181
D269
Y308
E103
D104
d
des2.1.1 (PDB ID:6WI3)
H183
D181
D269
Y308
F210
D104

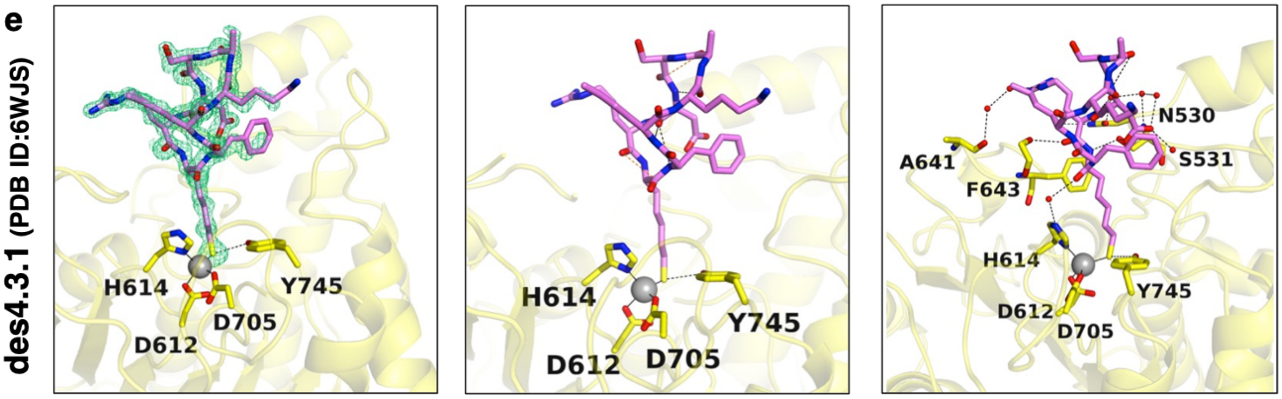

Supplementary Figure 3. Crystal structure of different HDAC variants and their interactions with the protein. Details of crystal structures of different designed macrocycles: (a) des1.1.0, (b) des1.1.1, (c) des1.1.2, (d) des2.1.1, and (e) des4.3.1. Polder omit map are contoured at 3.0σ for all the designs but Des4.3.1 (4.0σ) showing the binding of the peptides in the active site of HDAC2 (HDAC6 for des4.3.1). Incomplete electron density in some residues of the peptide suggests some measure of conformational disorder for these inhibitor residues. The catalytic Zn^{2+} ion (grey sphere) is coordinated in tetrahedral fashion by three protein residues (two Asp and one His) and the thiolate side chain of SHA, which also forms a hydrogen bond with a Tyr near the active site. Metal coordination and hydrogen bond interactions are shown as dashed black lines. Water molecules are shown as red spheres. Chains B, A, A, and A were used due to lower B-factor for des1.1.0, 1.1.1, 1.1.2, and 2.1.1 respectively.

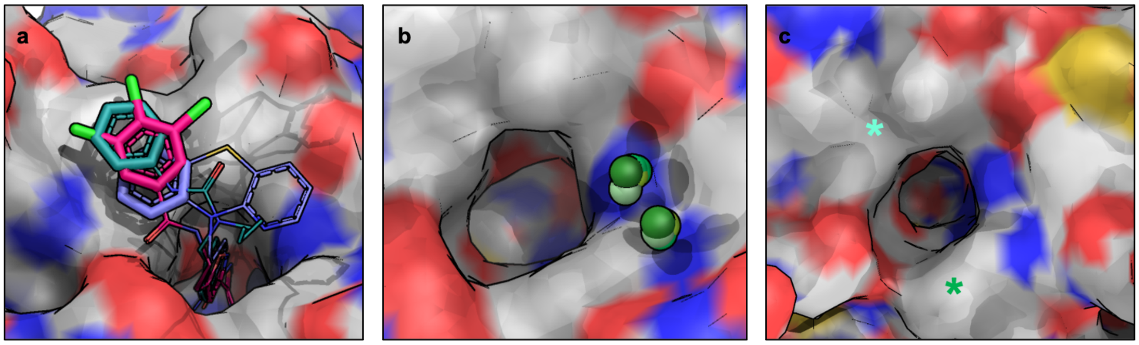

Supplementary Figure 4. Structural analysis of HDAC structures suggests new design directions for enhanced binding. (a) An aromatic ring is observed in a conserved position in many of HDAC binders (purple: PDB ID 5W5K, green: PDB ID 5G0I, pink: PDB ID 6R0K). (b) Conserved water molecules at the binding pocket of HDAC2 (from PDB IDs 5IWG, 4LY1, 6GO3). (c) The two asterisks show the hydrophobic patches immediately adjacent to the active site pocket of HDAC2.

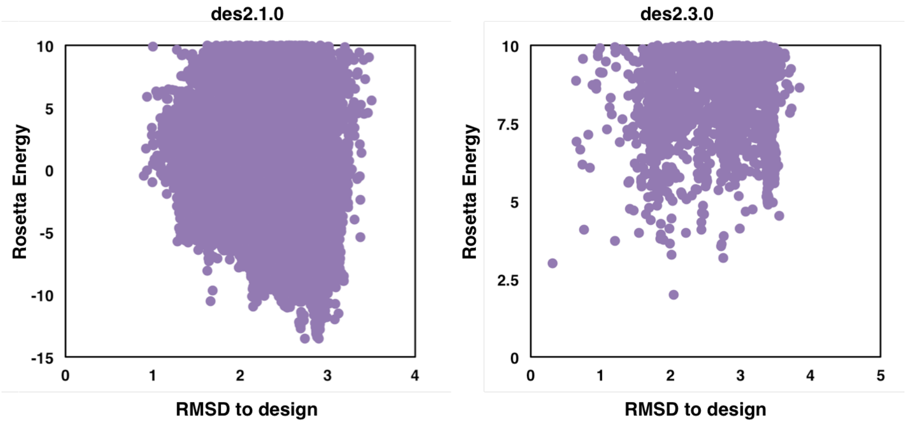

Supplementary Figure 5. Computational conformational sampling of designs from method 2 suggests peptide flexibility. Representative computational conformational sampling of two designs from design method 2. Source Data are provided as a Source Data file.

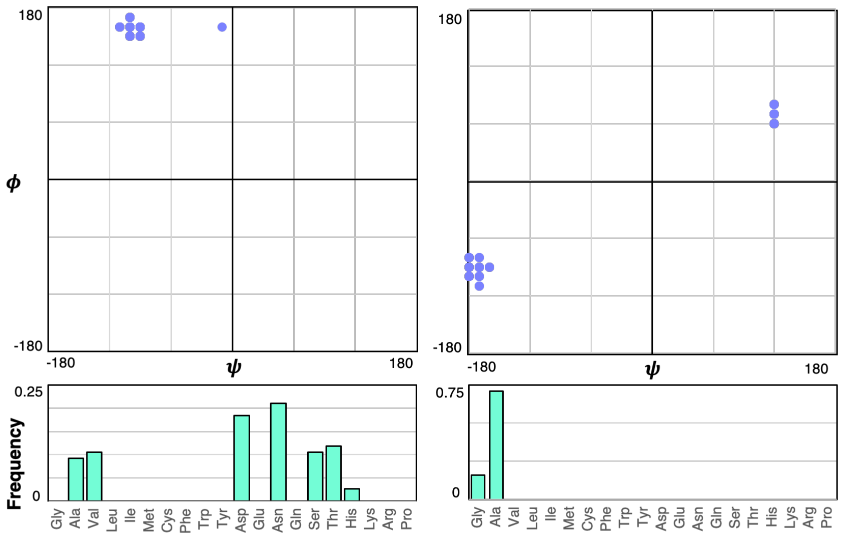

Supplementary Figure 6. Exhaustive sampling of residues around SHA anchor in design method 3 converge to few torsions and amino acids. Torsion and amino acid distribution of the top selected sampled peptides from method 3 for residue right before SHA (left) and the one right after SHA (right).

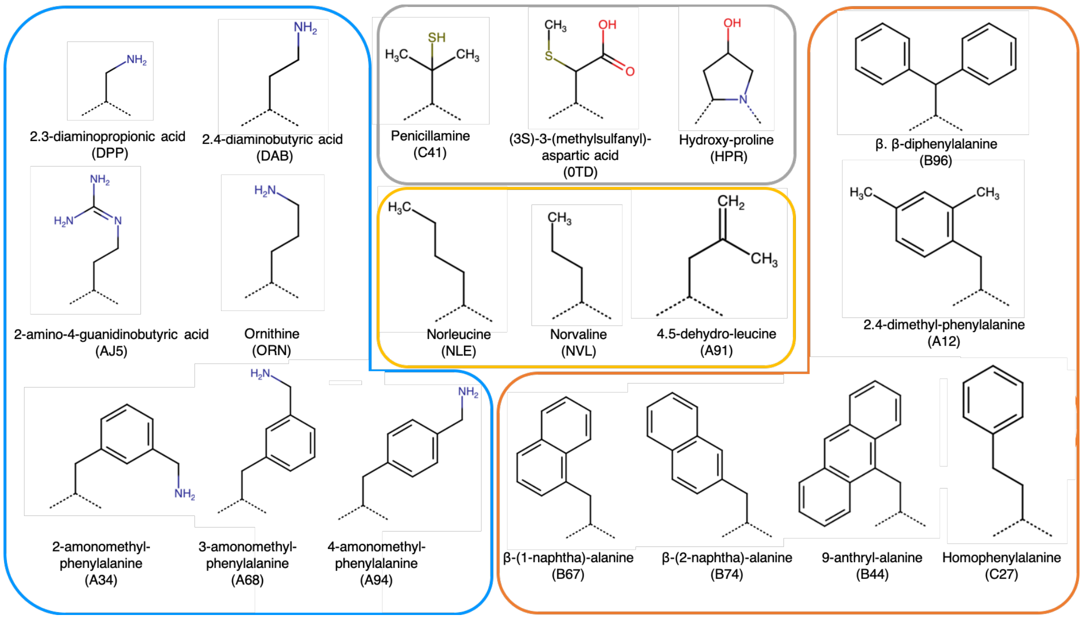

Supplementary Figure 7. Noncanonical amino acids used in our design. Chemical structure, name, and Rosetta 3 letter code of the noncanonical amino acids used in design method 4. The amino acids are grouped based on the properties of their sidechain into 4 groups: positively charged (blue), small hydrophobic (yellow), aromatic (orange), and others (grey).

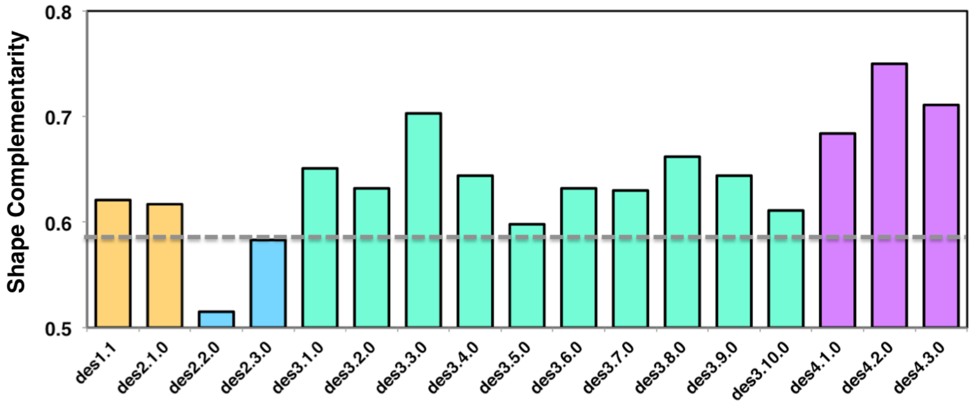

Supplementary Figure 8. Design methods 3 and 4 overall show higher shape complementarity between the peptide and protein pocket. Comparison of the selected designs from methods 1-4 for shape complementarity, an in silico binding metrics

representative of how well the macrocycle matches the binding interface in terms of shape. The dashed line shows the average value for method 1 and 2. Macrocycles designed using method3 and 4 in general showed better shape complementarity value; this better overall performance reflects in the macrocycles selected to test as well -shown in this figure-

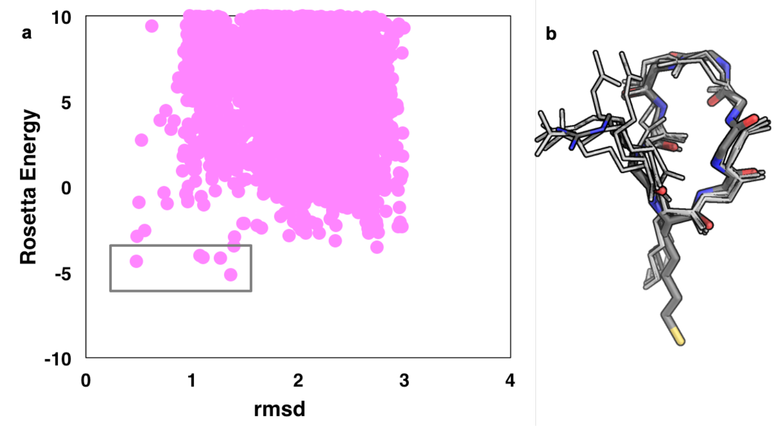

Supplementary Figure 9. Conformational sampling of D-Arg→ L-Arg mutation in des4.3.1. (a) Conformational sampling of des431: D-Arg→ L-Arg shows that Rosetta can capture the observed conformation in the crystal structure. (b) The peptide structure from crystal structure (PDB ID: 6WSJ, dark grey sticks) is overlayed with top 5 best scoring structures shown as light grey lines.

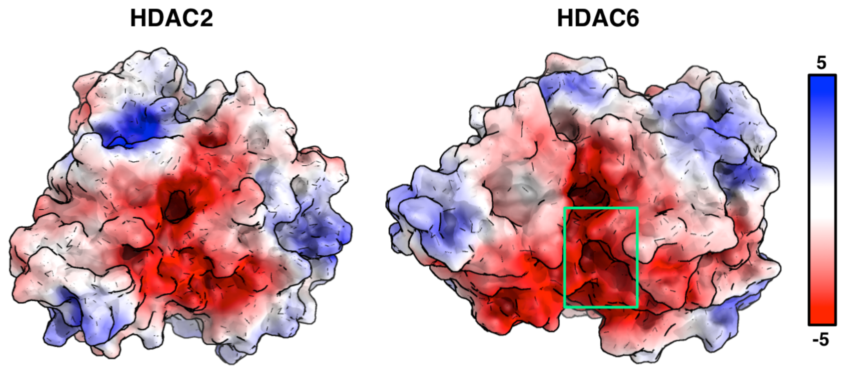

Supplementary Figure 10. HDAC2 and HDAC6 have different electrostatic surface potential. Electrostatic surface potential of HDAC2 and HDAC6 contoured at 1/-1 kT/e. The green box shows the relative position of des4.3.1 D-Arg-4 interaction with HDAC6.

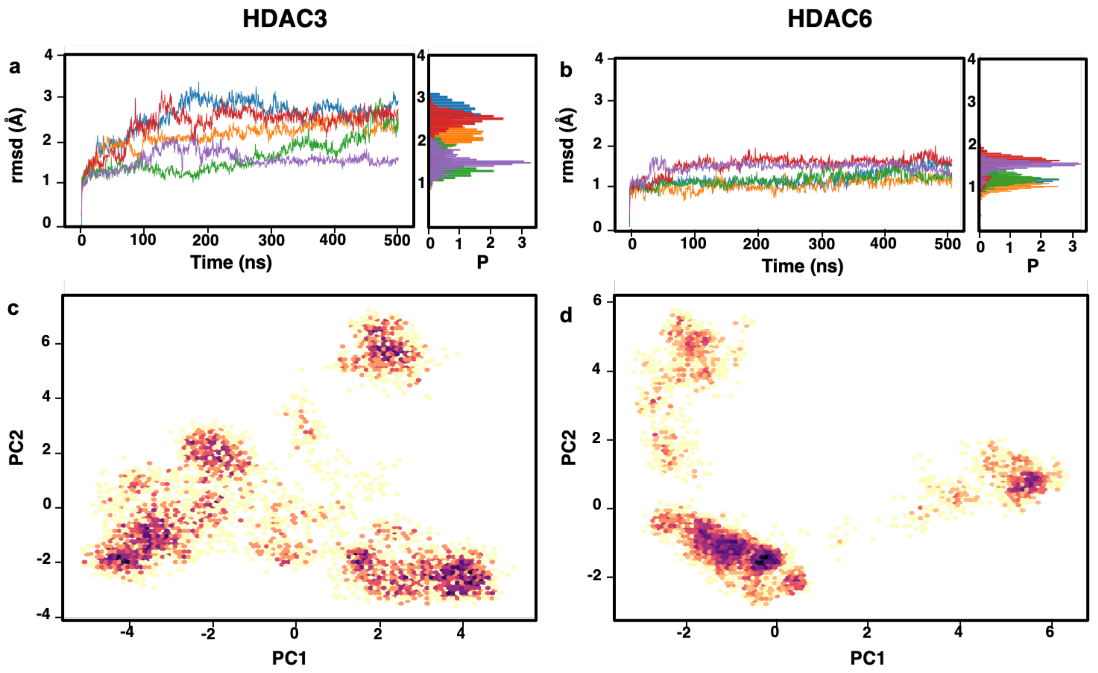

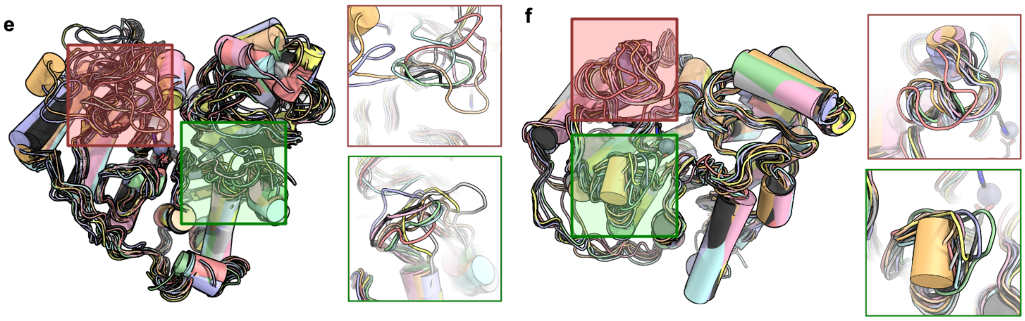

Supplementary Figure 11. MD simulations display structural flexibility in different HDACs. Panels a, b show RMSD to the starting conformation of the MD trajectories (5x100ns) for HDAC3 and HDAC6. The RMSD traces (left panels) and histograms (right panels) are shown for each trajectory with corresponding colors. Panels c and d show 2D histograms of the combined MD trajectories projected onto the first two principal components calculated from the phi and psi dihedral angles. Panels e and f show overlay of representative members of different conformational clusters observed during simulation.

<u>des1.1.0</u>

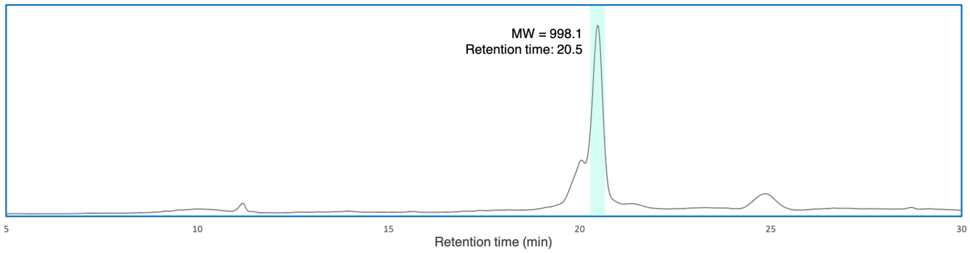

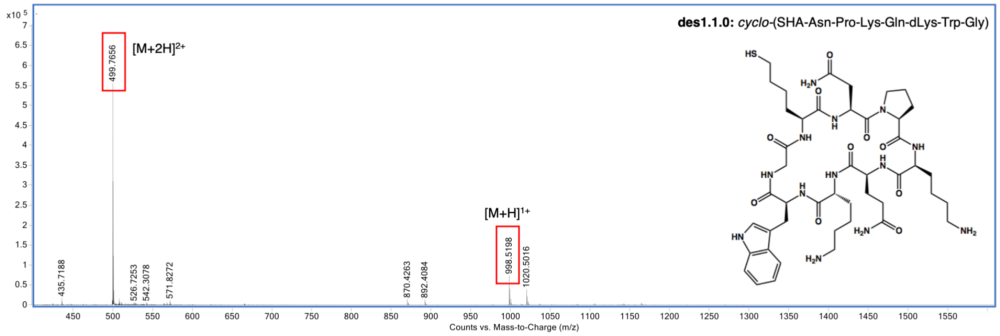

<u>des1.1.1</u>

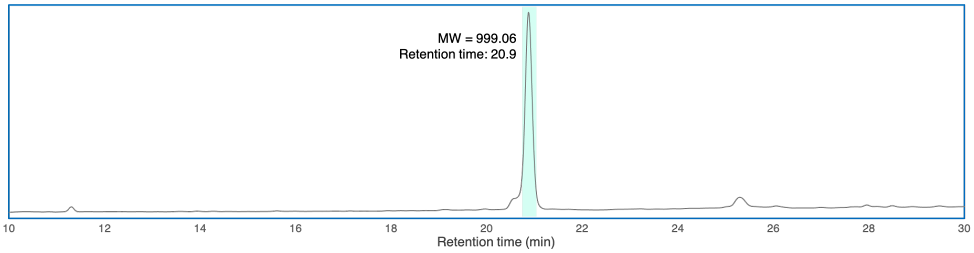

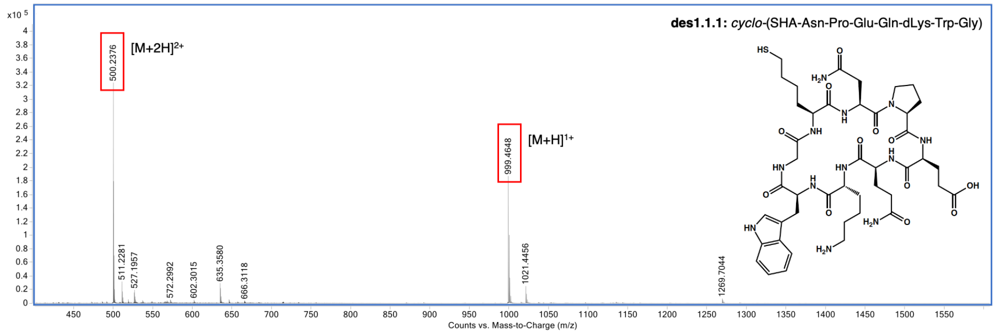

<u>des1.1.2</u>

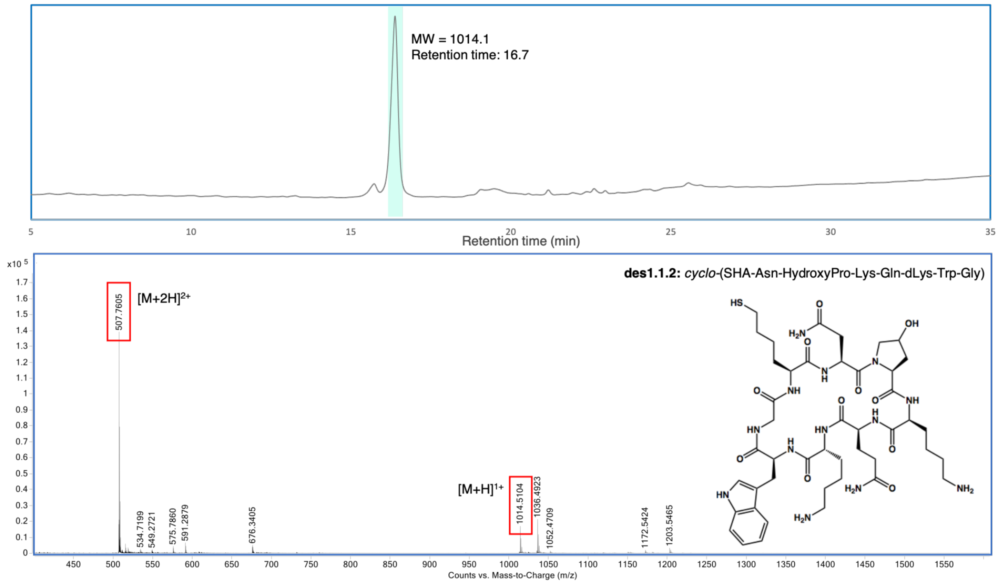

des2.1.0

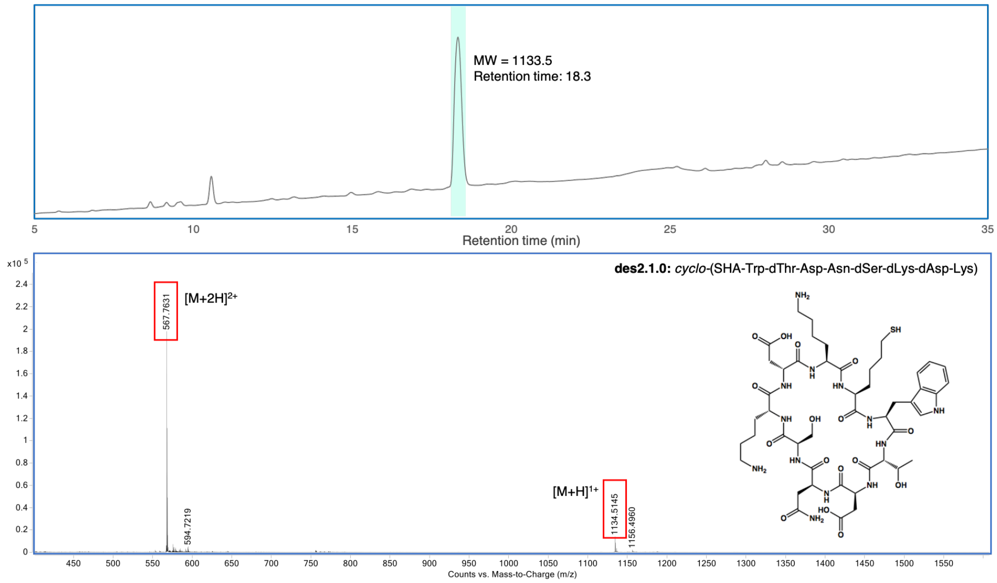

des2.1.1

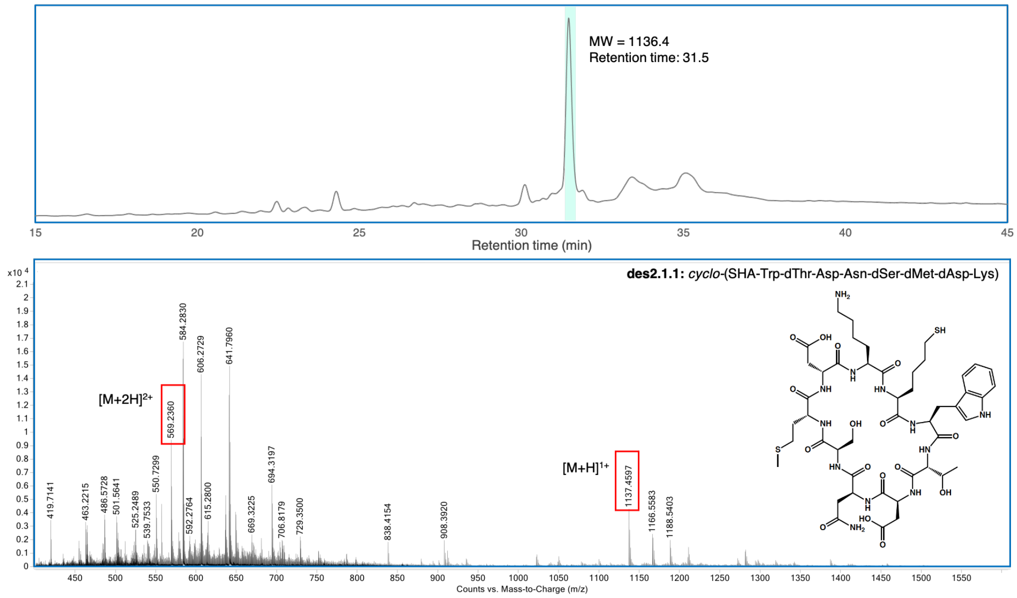

des2.2.0

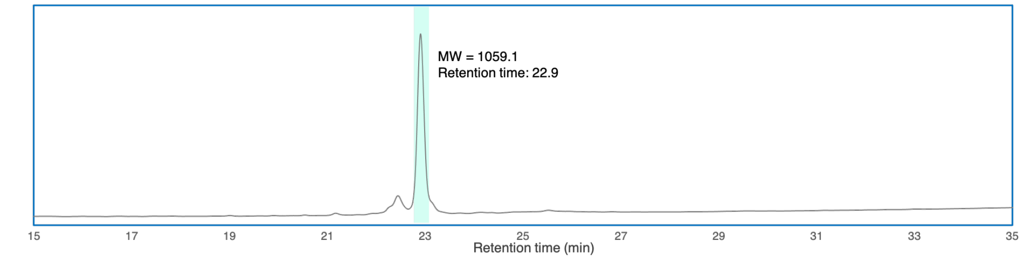

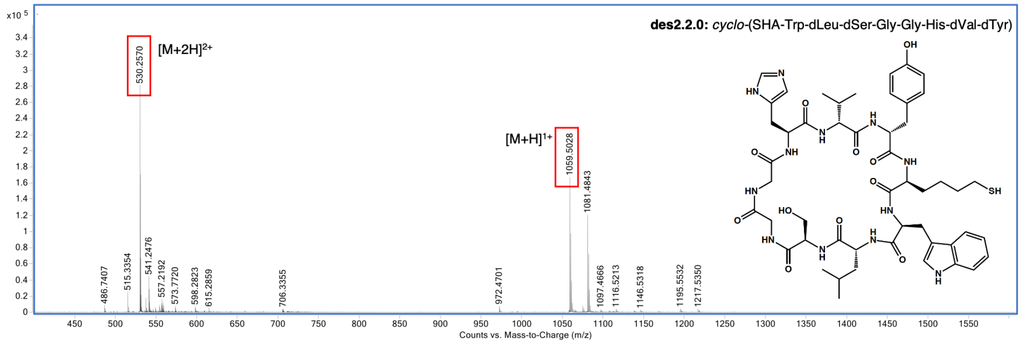

des3.1.0

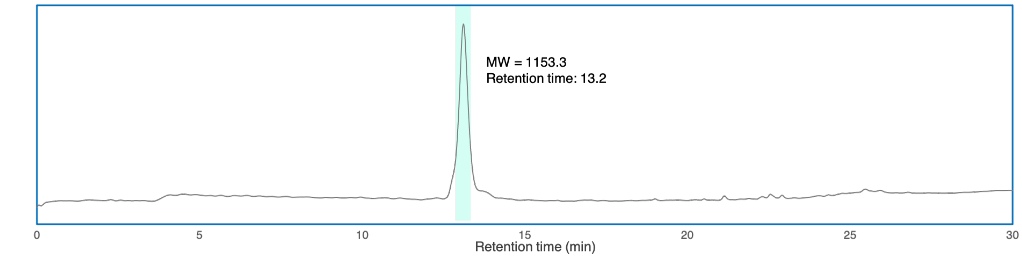

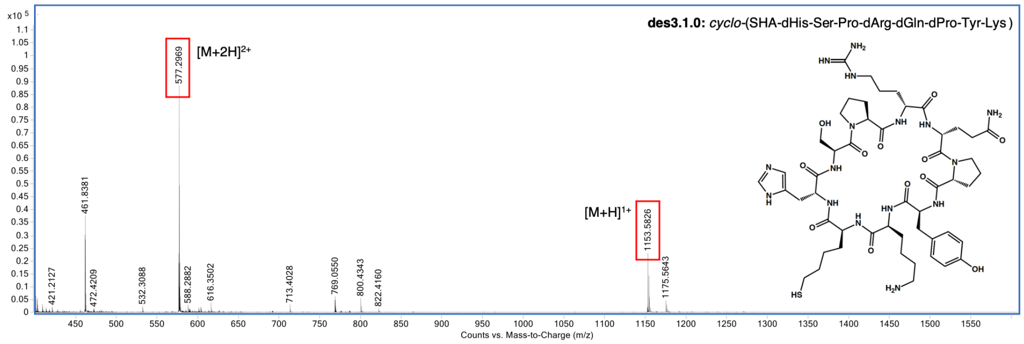

des3.2.0

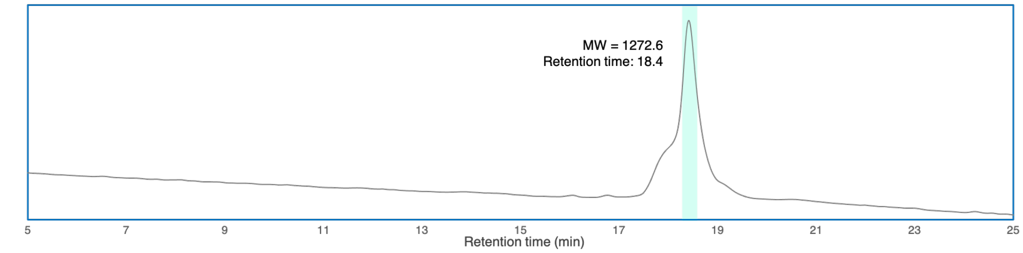

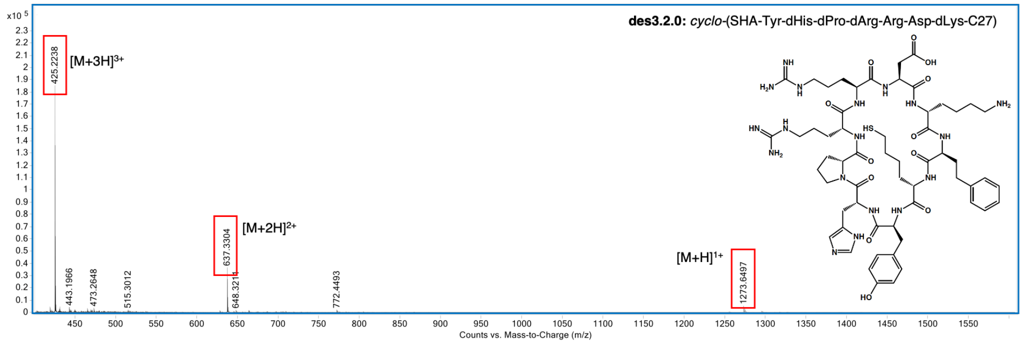

des3.2.1

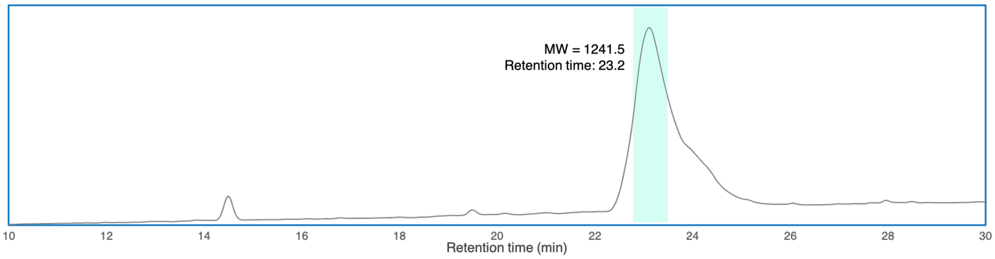

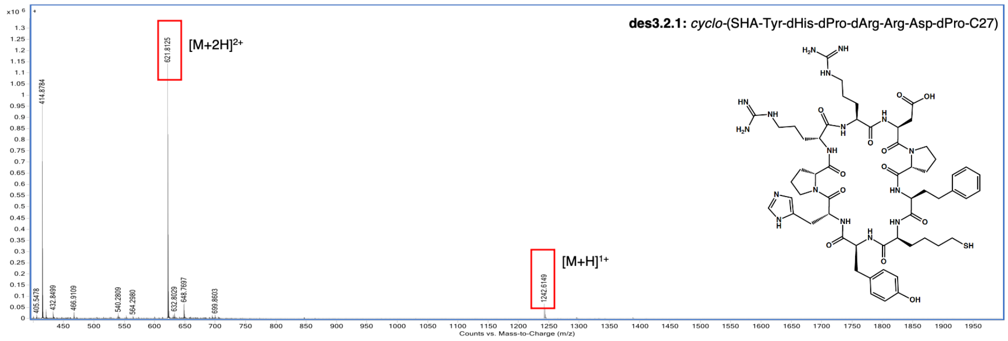

des3.3.0

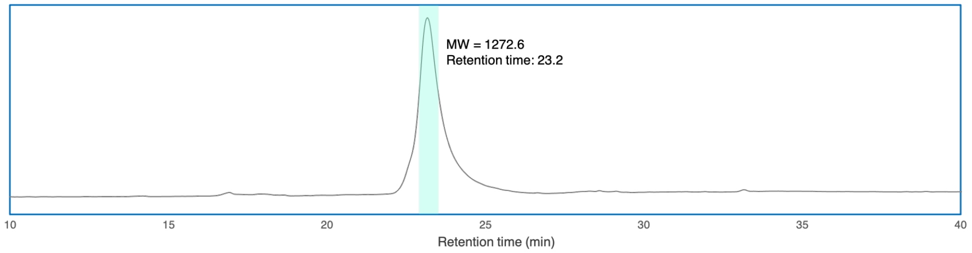

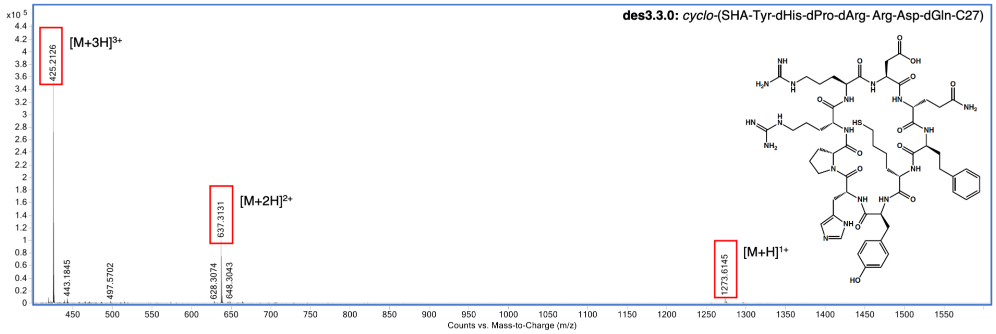

des3.3.1

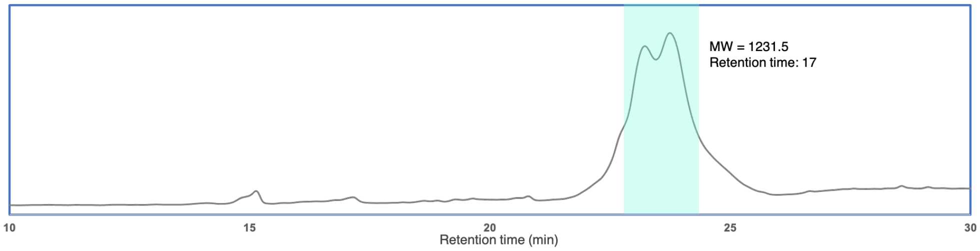

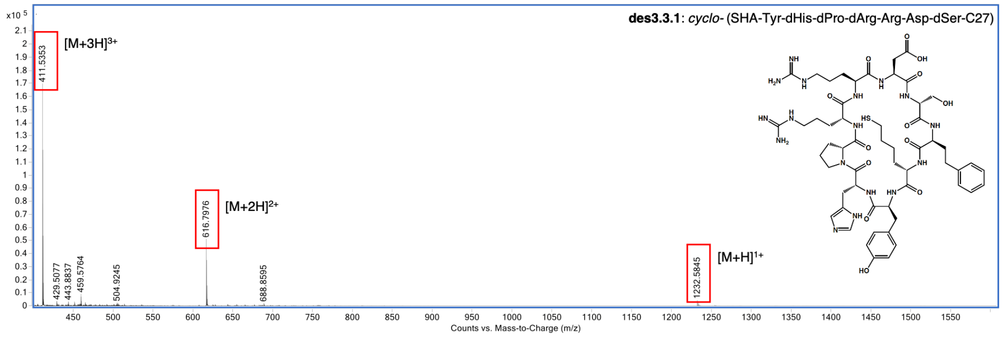

des3.3.2

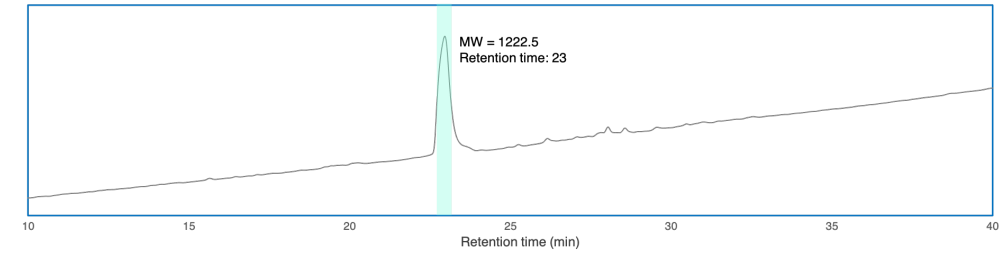

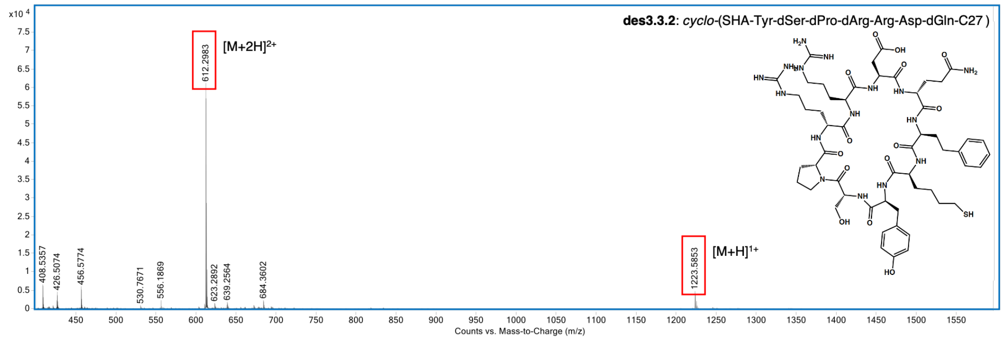

des4.1.0

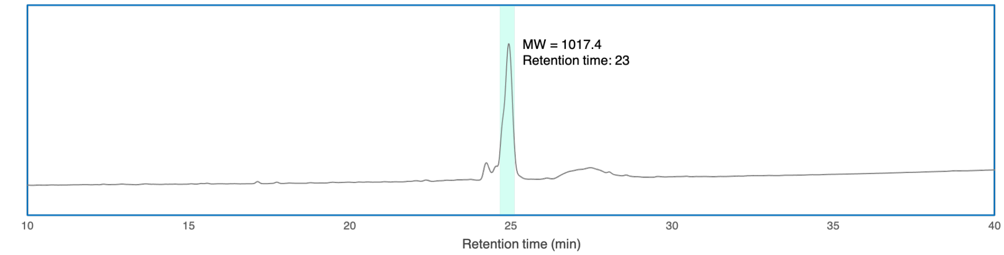

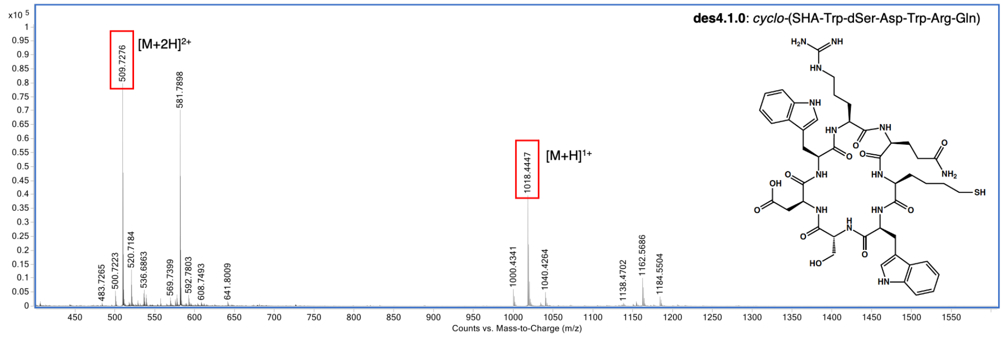

des4.2.0

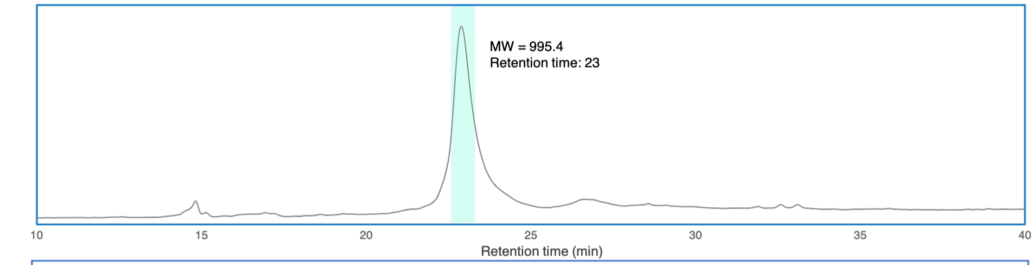

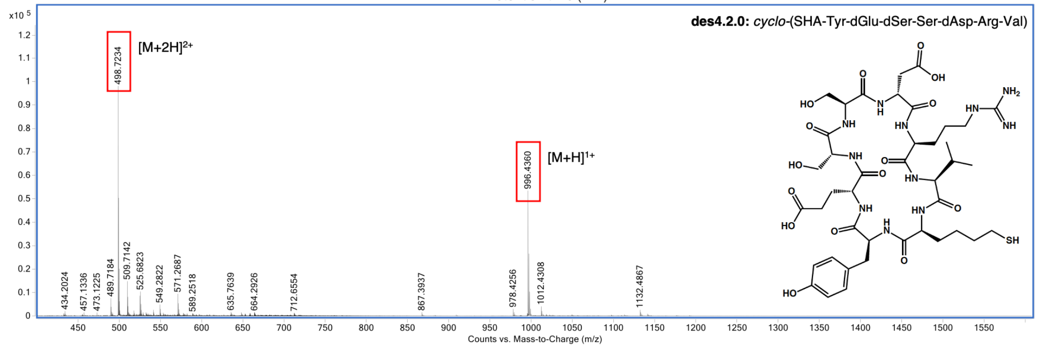

des4.3.1

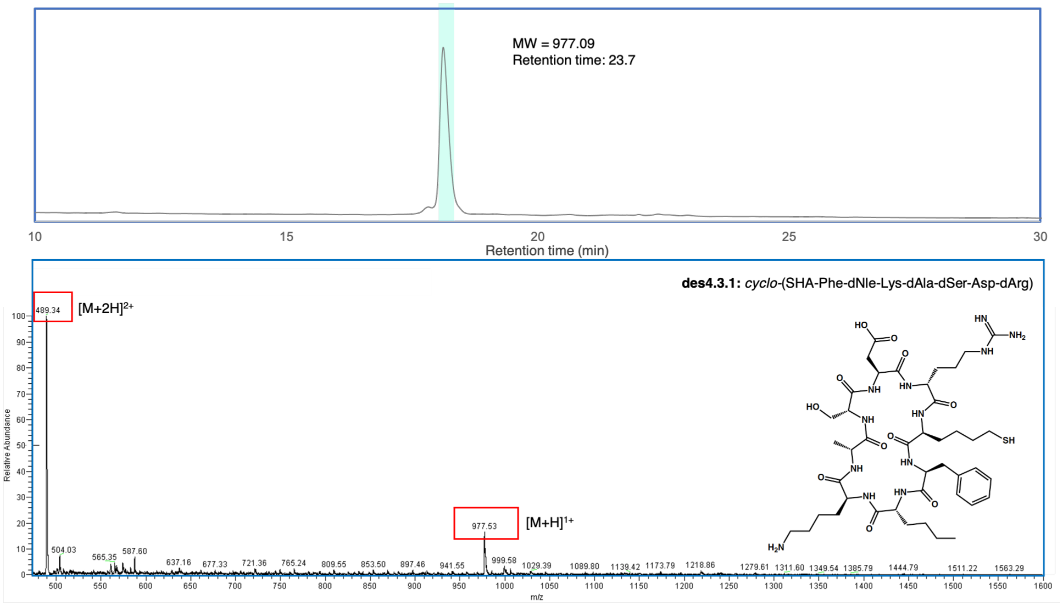

Supplementary Figure 12. LD-MS data of peptides used in HDAC inhibition assay. All runs were from 5-95% at 2%/min, with A = H2O w/ .1% TFA, and B = 90% ACN/10% H2O w/ .1% TFA. The spectra were collected at 220nm. The green shades are the fraction that was collected. Source data are provided in Source Data file.

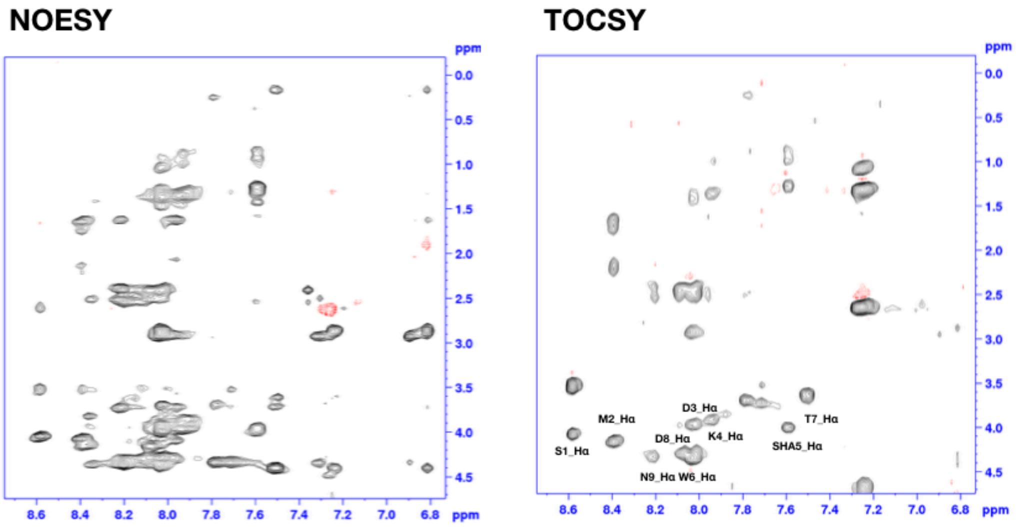

Supplementary Figure 13. NOESY and TOCSY NMR spectra of design2.1.1. Important atoms are labeled.

Supplementary Tables

Supplementary Table 1. Binding affinity of some of most potent peptide-based and peptide-like HDAC inhibitors. All values are IC_{50} [nM], except for Apicidin for which EC_{50} was reported. Orange cells are those with highest potency.

Name	Type and reference	Class I				Class IIa	Class IIb
		HDAC1	HDAC2	HDAC3	HDAC8	HDAC4	HDAC6
Cyl-2	tetrapeptide with ncAA, covalent inhibitor[1]	0.70 ± 0.45				NA	40,000 ± 11,000
Trapoxin B	tetrapeptide with ncAA, covalent inhibitor[1]	**0.11 ± 0.01**				**0.3 ± 0.03**	360 ± 160
Apicidin 39 (highest potency)	Tetrapeptide with ncAA[2]		120	43	575		
Romidepsin	Depsipeptide, activated upon reduction[3]	1.6 ± 0.9	**3.9 ± 2.7**			25 ± 7.3	790 ± 110
4a (HDAC1 selective)	$\alpha3\beta$ peptide from library[4]	20		62 ± 7	4920		>10,000
3c (Highest potency)	$\alpha3\beta$ peptide from library[4]	2 ± 1.4		18 ± 3	133		31 ± 2
23 (HDAC6 selective)	$\alpha3\beta$ peptide from library[4]	122 ± 15		164 ± 4	244 ± 9		39 ± 3
des3.3.0	Computational design (this study)	4.2	9.1	6.4	244		11.6
des4.2.0	Computational design (this study)	58.2	214	71.2	370		**4.4**

Supplementary Table 2. IC_{50} values of selected macrocyclic designs against HDACs 1,2,3,6,8. FF column indicates how likely these peptides are to fold into the designed model based on Rosetta's conformational sampling. Darker blue indicates highest probability of folding and red means not folded.

	HDAC1 (IC_{50})	HDAC2 (IC50)	HDAC3 (IC50)	HDAC6 (IC50)	HDAC8 (IC50)	FF	Sequence
* des1.1.0	1.88E-07	2.89E-07	4.76E-08	2.48E-08	2.16E-06		SHA-Asn-Pro-Lys-Gln-dLys-Trp-Gly
des1.1.1	1.37E-07	1.92E-07	1.61E-07	3.24E-08	1.39E-06		SHA-Asn-Pro-Glu-Gln-dLys-Trp-Gly
des1.1.2	5.23E-07	9.30E-07	7.77E-08	2.16E-08	7.72E-06		SHA-Asn-Hpr-Lys-Gln-dLys-Trp-Gly
des2.1.0	2.55E-08	4.93E-08	5.62E-08	3.23E-08	3.99E-07		SHA-Trp-dThr-Asp-Asn-dSer-dLys-dAsp-Lys
* des2.1.1	8.83E-09	1.63E-08	1.30E-08	7.54E-09	3.52E-07	•	SHA-Trp-dThr-Asp-Asn-dSer-dMet-dAsp-Lys
des2.2.0	9.47E-06	1.00E-05	1.21E-05	1.38E-06	5.68E-06		SHA-Trp-dLeu-dSer-Gly-Gly-His-dVal-dTyr
des3.1.0	3.24E-07	1.43E-06	7.24E-07	1.34E-06	2.73E-06		SHA-dHis-Ser-Pro-dArg-dGln-dPro-Tyr-Lys
des3.2.0	9.83E-09	2.78E-08	9.53E-09	1.46E-08	1.80E-07		SHA-Tyr-dHis-dPro-dArg-Arg-Asp-dLys-C27
des3.2.1	ND	6.62E-08	2.68E-08	2.90E-08	1.20E-07		SHA-Tyr-dHis-dPro-dArg-Arg-Asp-dPro-C27
des3.2.2	ND	5.29E-08	2.37E-08	2.23E-08	2.57E-07		SHA-Tyr-dHis-dPro-dArg-Pro-Asp-dLys-C27
des3.2.3	ND	4.04E-08	1.92E-08	1.85E-08	3.56E-07		SHA-Tyr-dHis-dPro-dArg-Pro-Asp-dPro-C27
des3.2.4	ND	6.77E-08	9.62E-08	2.87E-08	1.59E-07		SHA-Tyr-dHis-dPro-dAsn-Lys-C27
des3.2.5	ND	4.61E-08	8.74E-08	1.76E-08	ND		SHA-Tyr-dHis-dPro-dArg-Lys-C27
des3.3.0	4.18E-09	**9.12E-09**	6.37E-09	1.16E-08	2.44E-07		SHA-Tyr-dHis-dPro-dArg- Arg-Asp-dGln-C27
§ des3.3.1	9.38E-09	2.48E-08	4.12E-09	1.93E-08	1.08E-07		SHA-Tyr-dHis-dPro-dArg-Arg-Asp-dSer-C27
des3.3.2	4.38E-09	5.70E-08	1.21E-08	1.97E-07	2.17E-07		SHA-Tyr-dSer-dPro-dArg-Arg-Asp-dGln-C27
des4.1.0	7.31E-08	9.73E-08	6.06E-08	2.32E-08	5.04E-07		SHA-Trp-dSer-Asp-Trp-Arg-Gln
des4.2.0	5.82E-08	2.14E-07	7.12E-08	**4.42E-09**	3.70E-07		SHA-Tyr-dGlu-dSer-Ser-dAsp-Arg-Val
des4.3.1	7.54E-07	1.47E-06	6.26E-07	1.71E-08	8.18E-08		SHA-Phe-dNle-Lys-dAla-dSer-Asp-dArg
SHA	4.94E-06	5.43E-06	5.58E-06	6.702E-07	1.00E-05		
TSA	7.00E-09	1.76E-08	2.80E-08	1.05E-09	6.60E-07		

* These peptide samples had some impurities that could not be fully removed.
§ This peptide showed two peaks during purification that converted to each other (~1:1)
• Denotes folding into another structure with RMSD > 1.5 from designed model
C27 = homophenylalanine, Hpr = hydroxyproline, Nle = norleucine
ND = not determined

Supplementary Table 3. IC_{50} values for des1.1.0 and its variants for HDAC2 and HDAC6. (Hpr = hydroxyproline). 2-Selectivity is fold potency for HDAC2 over HDAC6. Mutations are highlighted in cyan.

	Sequence	HDAC2 IC_{50}	HDAC6 IC_{50}	2-Selectivity
des1.1.0	SHA-Asn-Pro-Lys-Gln-dLys-Trp-Gly	2.9×10^{-7}	2.5×10^{-8}	0.03
des1.1.1	SHA-Asn-Pro-Glu-Gln-dLys-Trp-Gly	1.9×10^{-7}	3.2×10^{-8}	0.17
des1.1.2	SHA-Asn-Hpr-Lys-Gln-dLys-Trp-Gly	9.3×10^{-7}	2.2×10^{-8}	0.02

Supplementary Table 4. Initial values for HDAC2 IC_{50} of designed macrocycles with different design methods. All the peptides are N-to-C cyclized unless otherwise stated. 'd' before name of amino acids means they are in D chiral state.

Peptide name	peptide sequence	MW	HDAC2 activity
SHA	SHA	159	5 µM
des1.1.0	SHA-Asn-Pro-Lys-Gln-dLys-Trp-Gly	998	289 nM
des1.1.0-linear	Ac-SHA-Asn-Pro-Lys-Gln-dLys-Trp-Gly-NH2	1016	1 µM
des1.1.0-partial, linear	Ac-Gly-SHA-Asn-NH2	348	10 µM
des1.1.1	SHA-Asn-Pro-Glu-Gln-dLys-Trp-Gly	999	192 nM
des1.1.2	SHA-Asn-Hpr-Lys-Gln-dLys-Trp-Gly	1014	930 nM
des1.1.3	SHA-Asn-Pro-Lys-Dap-dLys-Trp-Gly	956	10-100 nM
des1.1.4	SHA-Asn-Pro-Lys-Ser-dLys-Trp-Gly	957	10-100 nM
des1.1.5	SHA-Asn-Pro-Lys-Gln-dLys-Asn-Gly	926	100 nM-1 µM
des1.1.6	SHA-Asn-Pro-Lys-Gln-dLys-Thr-Gly	913	100 nM-1 µM
des1.2.0	SHA-dLys-dNle-Glu-dAsn-dThr-Arg-dAla	972	100 µM
des1.3.0	SHA-dAsn-dAsp-Glu-dAsn-dAsn-Arg-dAla	973	1 µM
des1.4.0	SHA-Leu-Val-dAsn-Orn-dAsp-dGlu-Ser-Pro-Pro-Asp-Arg-dAla	1467	100 µM
des2.1.0	SHA-Trp-dThr-Asp-Asn-dSer-dLys-dAsp-Lys	1133	49.3 nM
des2.1.1	SHA-Trp-dThr-Asp-Asn-dSer-dMet-dAsp-Lys	1136	16.3 nM
des2.2.0	SHA-Trp-dLeu-dSer-Gly-Gly-His-dVal-dTyr	1059	100 nM-1 µM
des2.2.1	SHA-Trp-dLeu-dSer-Gly-Gly-His-Thr-dTyr	1061	100 nM-1 µM
des2.2.2	SHA-Trp-dLeu-dSer-dPro-Gly-His-dThr-dTyr	1101	500 nM
des2.3.0	SHA-Trp-Gly-Gly-Met-His-Gly-dTrp-dPhe	1118	100 nM-1 µM
des2.3.1	SHA-Trp-dPro-Gly-Met-His-dSer-dPhe	1002	100 nM-1 µM
des3.1.0	SHA-dHis-Ser-Pro-dArg-dGln-dPro-Tyr-Lys	1153	1.4 µM
des3.1.1	SHA-dHis-Ser-Pro-dArg-dGln-dPro-Trp-Lys	1175	100 nM-1 µM
des3.2.0	SHA-Tyr-dHis-dPro-dArg-Arg-Asp-dLys-C27	1273	28 nM
des3.2.1	SHA-Tyr-dHis-dPro-dArg-Arg-Asp-dPro-C27	1243	66 nM
des3.2.2	SHA-Tyr-dHis-dPro-dArg-Pro-Asp-dLys-C27	1215	58 nM
des3.2.3	SHA-Tyr-dHis-dPro-dArg-Pro-Asp-dPro-C27	1184	92 nM
des3.2.4	SHA-Tyr-dHis-dPro-dAsn-Lys-C27	961	54 nM
des3.2.5	SHA-Tyr-dHis-dPro-dArg-Lys-C27	1001	95 nM
des3.3.0	SHA-Tyr-dHis-dPro-dArg- Arg-Asp-dGln-C27	1273	9.1 nM
des3.3.1	SHA-Tyr-dHis-dPro-dArg-Arg-Asp-dSer-C27	1231	25 nM
des3.3.2	SHA-Tyr-dSer-dPro-dArg-Arg-Asp-dGln-C27	1223	57 nM
des3.4.0	SHA-Tyr-dAsn-dPro-dArg-Arg-Asp-dLys-C27	1250	10-100 nM
des3.5.0	SHA-dAla-Phe-dPro-Asp-dGln-Dap-dAsn-Dab	1014	>1 µM

des3.5.1	SHA-dAla-Phe-Pro-dAsp-Asp-Ser-dAsn-Dab	1005	>1 μM
des3.6.0	SHA-dHis-dAla-Tyr-dAsn-dAsn-Pro-dArg-Trp	1198	>1 μM
des3.7.0	SHA-Nlu-dSer-Trp-dSer-dAsp-Pro-Ala-C27	1076	>1 μM
des3.8.0	SHA-dHis-dAsn-dGlu-Leu-dAla-Pro-Arg-B74	1174	>1 μM
des3.9.0	SHA-Phe-Dap-dArg-dSer-dAla-Pro-Nlu-B74	1112	10-100 nM
des3.10.0	SHA-dArg-Ala-Pro-dLys-dGlu-dPhe-dArg-C27	1205	10-100 nM
des4.1.0	SHA-Trp-dSer-Asp-Trp-Arg-Gln	1017	100 nM
des4.2.0	SHA-Tyr-dGlu-dSer-Ser-dAsp-Arg-Val	995	200 nM
des4.3.1	SHA-Phe-dNle-Lys-dAla-dSer-Asp-dArg	977	350 nM

ncAAs used:

- B74:beta-(2-naphthyl)-alanine
- C27:Homophenylalanine
- Dab:2,4-diaminobutyric acid
- Dap:2,3-diaminopropionic acid
- Hpr:Hydropxy Proline
- Nlu: Norleucine
- Nva:Norvaline
- Orn:Ornithine
- SHA: 2S-2-amino-7-sulfanylheptanoic acid

Supplementary Table 5. IC_{50} values for des3.2.0 and its variants for HDAC2 and HDAC6. 2-Selectivity is defined as fold potency for HDAC2 over HDAC6. Mutations are highlighted in cyan.

	Sequence	HDAC2 IC_{50}	HDAC6 IC_{50}	2-Selectivity
des3.2.0	SHA-Tyr-dHis-dPro-dArg-Arg-Asp-dLys-C27	2.8×10^{-8}	1.5×10^{-8}	0.53
des3.2.1	SHA-Tyr-dHis-dPro-dArg-Arg-Asp-dPro-C27	6.6×10^{-8}	2.9×10^{-8}	0.44
des3.2.2	SHA-Tyr-dHis-dPro-dArg-Pro-Asp-dLys-C27	5.3×10^{-8}	2.2×10^{-8}	0.41
des3.2.3	SHA-Tyr-dHis-dPro-dArg-Pro-Asp-dPro-C27	4.0×10^{-8}	1.9×10^{-8}	0.47
des3.2.4	SHA-Tyr-dHis-dPro-dAsn-Lys-C27	6.8×10^{-8}	2.9×10^{-8}	0.43
des3.2.5	SHA-Tyr-dHis-dPro-dArg-Lys-C27	4.6×10^{-8}	1.8×10^{-8}	0.40

Supplementary Table 6. IC_{50} values for des4.3.1 and its variants for HDAC2 and HDAC6. 6-Selectivity is defined as fold potency for HDAC6 over HDAC2. Mutations are highlighted in cyan.

	Sequence	HDAC2 IC_{50}	HDAC6 IC_{50}	6-Selectivity
des4.3.1	SHA-Phe-dNle-Lys-dAla-dSer-Asp-dArg	1.5×10^{-6}	1.7×10^{-8}	88.2
des4.3.1.1	SHA-Phe-dNle-Lys-dAla-dSer-Asp-dSer	5.0×10^{-6}	1.6×10^{-7}	31.25
des4.3.1.2	SHA-Phe-dLys-Lys-dAla-dSer-Asp-dNle	NA	5.4×10^{-7}	> 100
des4.3.1.3	SHA-Phe-dNle-Lys-dAla-dSer-Ala-dArg	1.4×10^{-6}	1.0×10^{-8}	140

Supplementary Table 7. Rosetta interface metrics analysis for crystal structure of des1.1.0 and des4.3.1. We assume a relative error of 0.5 REU (Rosetta Energy Unit) for all calculations.

	**des1.1.0, model**	**des1.1.0, crystal-chain B**	**des4.3.1, crystal**
**ddg**	-11.1	-22.3	-17.0
**ddg_norepack**	-20.9	-28.5	-21.2
**dsasa**	0.4	0.4	0.4
**interface_buried_sasa**	768.6	825.4	712.1
**interface_contactcount**	34.9	41.6	24.9
**interface_hydrophobic_sasa**	570.9	589.5	531.1
**interface_polar_sasa**	204.6	237.1	184.0
**Shape complementarity**	0.6	0.8	0.7

Supplementary Table 8. Comparison of selective HDAC6 inhibitors in literature and des4.2.0. Values are obtained from Selleckchem HDAC6 selective inhibitor catalog (selleckchem.com).

**Compound**	**IC_{50} [nM]**	**selectivity**
CAY10603	0.002	>200-fold selectivity over other HDACs.
Tubacin	4	approximately 350-fold selectivity over HDAC1.
Rocilinostat (ACY-1215)	4.7	>10-fold more selective for HDAC6 than HDAC1/2/3 (class I HDACs) with slight activity against HDAC8, minimal activity against HDAC4/5/7/9/11
Nexturastat A	5	>190-fold selectivity over other HDACs
Tubastatin A	15	selective (1000-fold more) against all other isozymes except HDAC8 (57-fold more).
HPOB	56	HPOB is a potent, selective HDAC6 inhibitor with IC50 of 56 nM, >30-fold selectivity over other HDACs.
**des4.2.0**	4.4	80 fold selective for HDAC6 over HDAC8 and >10-fold selectivity for HDAC6 over HDACs1/2/3

Supplementary Table 9. NMR chemical shifts of design 2.1.1

	Hα	HN	Hβ	Hγ	Hδ	Hε	Others:
**S_1**	4.06	8.60	3.52				
**M_2**	4.16	8.40	1.65,1.75	2.52,2.14		1.99	
**D_3**	3.73	7.97	1.63,2.07				
**K_4**	3.94	7.95	1.35	0.99	0.88	2.93	
**SHA_5**	3.97	7.60	1.26	0.93	1.43	1.58	Hζ:2.54
**W_6**	4.40	8.05	2.89				Hε1:9.93<br>Hε3:7.25,<br>Hε2:7.14<br>Hδ1:6.82<br>Hη2:6.90<br>Hζ3:6.51
**T_7**	4.41	7.51	3.63	0.17			
**D_8**	4.30	8.10	2.42,2.53				
**N_9**	4.37	8.22	2.41,2.54				Hδ1:6.67<br>Hδ2:7.37

Supplementary Table 10. Crystallographic data[a]

Structures	**des1.1.0 (HDAC2)** PDB ID: 6WHN	**des1.1.1 (HDAC2)** PDB ID: 6WHO	**des1.1.1' (HDAC2)** PDB ID: 6WHQ	**des1.1.2 (HDAC2)** PDB ID: 6WHZ	**des2.1.1 (HDAC2)** PDB ID: 6WI3	**des4.3.1 (HDAC6)** PDB ID: 6WSJ
**Data collection**						
Space group	$P2_12_12_1$	$P2_12_12_1$	$P2_12_12_1$	$P2_12_12_1$	$P2_12_12_1$	$P\ 22_12_1$
a, *b*, *c* (Å)	92.34, 97.66, 138.98	92.54, 97.48, 138.97	92.44, 97.30, 138.63	92.47, 94.80, 138.81	92.16, 97.59, 138.07	51.5, 84.0, 94.4
α, *β*, *γ* (°)	90, 90, 90	90, 90, 90	90, 90, 90	90, 90, 90	90, 90, 90	90.0, 90.0, 90.0
R_{merge}[b]	0.098 (0.731)	0.178 (1.417)	0.185 (1.652)	0.290 (2.026)	0.227 (1.676)	0.126 (0.619)
R_{pim}[c]	0.039 (0.309)	0.070 (0.583)	0.073 (0.647)	0.112 (0.792)	0.089 (0.656)	0.079 (0.413)
$I/\sigma(I)$	17.5 (1.7)	10.5 (1.4)	10.0 (1.2)	7.6 (1.1)	8.7 (1.2)	9.8 (3.8)
$CC_{1/2}$[d]	0.987 (0.539)	0.995 (0.541)	0.996 (0.574)	0.991 (0.548)	0.993 (0.516)	0.994 (0.845)
Completeness (%)	98.4 (89.6)	100 (100)	100 (100)	100 (100)	100 (100)	100 (100)
Redundancy	7.0 (5.7)	7.3 (6.8)	7.4 (7.5)	7.3 (7.4)	7.4 (7.5)	6.4 (5.8)
**Refinement**						
Resolution (Å)	43.82 -1.54 (1.68 – 1.54)[a]	48.74 – 2.20 (2.25 - 2.20)	48.65 – 2.35 (2.42 - 2.35)	47.90 – 2.90 (3.08 – 2.90)	48.80 – 2.35 (2.42 – 2.35)	62.76 – 1.70 (1.76 – 1.70)
No. reflections	183886	64445	52747	27684	52926	45672

R_{work} / R_{free} (%)[e]	16.2 / 19.2 (26.2 / 29.7)	18.4 / 22.0 (28.8 / 32.1)	19.5 / 24.1 (31.3 / 35.5)	22.7 / 28.1 (36.6 / 43.4)	19.2 / 25.3 (29.2 / 37.0)	15.4 / 17.5 (18.0 / 22.3)
**Number of atoms[f]**						
Protein	10058	9579	9462	8958	9385	3057
Ion /Ligand	211	210	291	186	205	79
Water	84	83	82	75	84	238
Ramachandran[g] Favored/allowed Outlier (%)	98.47/1.53 00.00	98.55/1.45 00.00	97.37/2.63 00.00	94.98/5.02 00.00	97.54/2.46 00.00	97.13/2.87 00.00
**R.M.S. deviations**						
Bond lengths (Å)	0.012	0.002	0.002	0.002	0.001	0.007
Bond angles (°)	1.2	0.5	0.5	0.4	0.5	1.2
**$B_{factors}$ (Å^2)[g]**						
Protein	23	41	51	65	46	12
Ion /Ligand	40	54	58	73	60	16
Water	35	43	49	40	43	21

1. Data were collected from one crystal per condition.
2. [a]Values in parentheses refer to the highest-resolution shell indicated. [b]$R_{merge} = \sum_{hkl}\sum_i|I_{i,hkl} - \langle I \rangle_{hkl}|/\sum_{hkl}\sum_i I_{i,hkl}$, where $\langle I \rangle_{hkl}$ is the average intensity calculated for reflection *hkl* from replicate measurements. [c]$R_{p.i.m.} = (\sum_{hkl}(1/(N-1))^{1/2}\sum_i|I_{i,hkl} - \langle I \rangle_{hkl}|)/\sum_{hkl}\sum_i I_{i,hkl}$, where $\langle I \rangle_{hkl}$ is the average intensity calculated for reflection *hkl* from replicate measurements and N is the number of reflections. [d]Pearson correlation coefficient between random half-datasets. [e]$R_{work} = \sum||F_o| - |F_c||/\sum|F_o|$ for reflections contained in the working set. $|F_o|$ and $|F_c|$ are the observed and calculated structure factor amplitudes, respectively. R_{free} is calculated using the same expression for reflections contained in the test set held aside during refinement. [f]Per asymmetric unit. [g]Calculated with MolProbity.
