# Supplementary material for: Anchor extension: a structure-guided approach to design cyclic peptides targeting enzyme active sites
Source: Nat Commun. 2021 Jun 7;12:3384. doi: 10.1038/s41467-021-23609-8 (PMC8185074; doi:10.1038/s41467-021-23609-8)
Supplement: Supplementary file 3 — Description of Additional Supplementary Files [file 41467_2021_23609_MOESM3_ESM.docx]

Description of Additional Supplementary Files

Title: Supplementary data file 1

Description: Includes all the codes used for each design method as well as detailed description of how to run them.

Title: Supplementary data file 2

Description: Includes all the sequences for the peptides described in the paper, their molecular weights, and their initial activity
