# Supplementary material for: Anchor extension: a structure-guided approach to design cyclic peptides targeting enzyme active sites
Source: Nat Commun. 2021 Jun 7;12:3384. doi: 10.1038/s41467-021-23609-8 (PMC8185074; doi:10.1038/s41467-021-23609-8)
Supplement: Supplementary file 6 — Reporting Summary [file 41467_2021_23609_MOESM6_ESM.pdf]

nature research

Corresponding author(s): David Baker

Last updated by author(s): Apr 24, 2021

# Reporting Summary

Nature Research wishes to improve the reproducibility of the work that we publish. This form provides structure for consistency and transparency in reporting. For further information on Nature Research policies, see our Editorial Policies and the Editorial Policy Checklist.

## Statistics

For all statistical analyses, confirm that the following items are present in the figure legend, table legend, main text, or Methods section.

| n/a | Confirmed | |
|---|---|---|
| x | ☐ | The exact sample size (*n*) for each experimental group/condition, given as a discrete number and unit of measurement |
| x | ☐ | A statement on whether measurements were taken from distinct samples or whether the same sample was measured repeatedly |
| x | ☐ | The statistical test(s) used AND whether they are one- or two-sided<br>*Only common tests should be described solely by name; describe more complex techniques in the Methods section.* |
| x | ☐ | A description of all covariates tested |
| x | ☐ | A description of any assumptions or corrections, such as tests of normality and adjustment for multiple comparisons |
| x | ☐ | A full description of the statistical parameters including central tendency (e.g. means) or other basic estimates (e.g. regression coefficient) AND variation (e.g. standard deviation) or associated estimates of uncertainty (e.g. confidence intervals) |
| x | ☐ | For null hypothesis testing, the test statistic (e.g. *F*, *t*, *r*) with confidence intervals, effect sizes, degrees of freedom and *P* value noted<br>*Give P values as exact values whenever suitable.* |
| x | ☐ | For Bayesian analysis, information on the choice of priors and Markov chain Monte Carlo settings |
| x | ☐ | For hierarchical and complex designs, identification of the appropriate level for tests and full reporting of outcomes |
| x | ☐ | Estimates of effect sizes (e.g. Cohen's *d*, Pearson's *r*), indicating how they were calculated |

*Our web collection on statistics for biologists contains articles on many of the points above.*

## Software and code

Policy information about availability of computer code

| | |
|---|---|
| Data collection | Rosetta software was used to generate computational design models and scores. All codes are available in the Methods section and supplementary data file 1. Rosetta is a free academic software. The provided code is updated to work with Rosetta release version 3.10.<br>The codes related the design methods can also be accessed through our github repository: https://github.com/ParisaH-Lab/publications.git, Peptide_HDACBinders folder.<br>GROMACS 2016.1 was used to generate MD results. |
| Data analysis | Python (3.0) and python pandas were used to analyze the scores generated in Rosetta.<br>GraphPad Prism 4.01 was used to analyze inhibition data.<br>imosflm 7.2.2, aimless 0.7.2 on CCP4 version 7.0.066, Phenix version 1.13-2998 and Coot version 0.9.5 were used for crystal structure indexing and refinement.<br>Sparky (3.114) was used for NMR assignment and Rosetta 3.10 was used to generate NMR models.<br>PyMOL 2.3.0 licensed to Institute for Protein Design was used for structure visualization and image generation.<br>Avogadro 1.2.0 and AMBER 12 with amber ff12sb are used for SHA conformation generation and sampling. |

For manuscripts utilizing custom algorithms or software that are central to the research but not yet described in published literature, software must be made available to editors and reviewers. We strongly encourage code deposition in a community repository (e.g. GitHub). See the Nature Research guidelines for submitting code & software for further information.

# Data

Policy information about availability of data

All manuscripts must include a data availability statement. This statement should provide the following information, where applicable:

- Accession codes, unique identifiers, or web links for publicly available datasets
- A list of figures that have associated raw data
- A description of any restrictions on data availability

Conformational sampling was done with the Rosetta simple_cycpep_predict application and peptide design was carried out with the rosetta_scripts application, both of which are included in the Rosetta software suite. The Rosetta software suite is available free of charge to academic users and can be downloaded from http://www.rosettacommons.org. Raw data of score and rmsd for the conformational sampling plots presented in the main text and supplementary information are provided in Source Data file. Instructions and inputs for running these applications, and all other data and code necessary to support the results and conclusion are provided in extended data file. The design scripts are also available in our github repository (https://github.com/ParisaH-Lab/publications.git).

All the structures presented here are deposited in PDB with accession codes 6WHN (http://doi.org/10.2210/pdb6WHN/pdb), 6WHO (http://doi.org/10.2210/pdb6WHO/pdb), 6WHQ (http://doi.org/10.2210/pdb6WHQ/pdb), 6WHZ (http://doi.org/10.2210/pdb6WHZ/pdb), 6WI3 (http://doi.org/10.2210/pdb6WI3/pdb), 6JSW (http://doi.org/10.2210/pdb6JSW/pdb). The raw data for HDAC inhibition assays (presented in Figures 2-5 and table S2) are available as a Source Data File. HPLC traces of all peptides are also available as a Source Data File.

# Field-specific reporting

Please select the one below that is the best fit for your research. If you are not sure, read the appropriate sections before making your selection.

☒ Life sciences ☐ Behavioural & social sciences ☐ Ecological, evolutionary & environmental sciences

For a reference copy of the document with all sections, see nature.com/documents/nr-reporting-summary-flat.pdf

# Life sciences study design

All studies must disclose on these points even when the disclosure is negative.

| | |
|---|---|
| Sample size | Backbone generation: To generate peptide backbones, we created around 10,000 structures. We stopped backbone generation at this range as the best binders started to converge in structure and sequence based on visual inspection of the data.<br>Conformational sampling: 10-100 peptides (depending on design method) from each round that passed our computational metrics threshold were selected for computational conformational sampling.<br>Experimental testing: Any peptide that passed this criteria moved on for experimental characterization (very few did). We also picked peptides that had the best computational interface metrics for downstream experimental testing. Overall, we performed initial testing on 42 peptides and full HDAC profiling was performed on 19 best designs. |
| Data exclusions | For one of IC50 calculations, a point was removed from des4.2.0_t1, HDAC8 assay as it was an outlier in the plot. |
| Replication | The inhibition assays were repeated in two independent replicates. All attempts at replication were successful, resulting in data within experimental error of one other with low standard deviation. All raw data are available in Supplementary file 1.<br>For structural studies using NMR and X-ray crystallography replication is not used. |
| Randomization | All the peptides tested in this paper were selected based on their computational score using a threshold and HDAC inhibition assay was performed on all of the ones that passed the computational conformational sampling and interface metrics, thus no randomization was performed. |
| Blinding | For our final HDAC activity assays, the Reaction Biology group who performed the assays was blind to the preliminary activity test performed and to computational interface metrics. Additionally both groups who performed crystallization and structure refinement were blind to the designed model of the peptides at the interface. |

# Reporting for specific materials, systems and methods

We require information from authors about some types of materials, experimental systems and methods used in many studies. Here, indicate whether each material, system or method listed is relevant to your study. If you are not sure if a list item applies to your research, read the appropriate section before selecting a response.

## Materials & experimental systems

| n/a | Involved in the study |
|---|---|
| ☒ | ☐ Antibodies |
| ☒ | ☐ Eukaryotic cell lines |
| ☒ | ☐ Palaeontology and archaeology |
| ☒ | ☐ Animals and other organisms |
| ☒ | ☐ Human research participants |
| ☒ | ☐ Clinical data |
| ☒ | ☐ Dual use research of concern |

## Methods

| n/a | Involved in the study |
|---|---|
| ☒ | ☐ ChIP-seq |
| ☒ | ☐ Flow cytometry |
| ☒ | ☐ MRI-based neuroimaging |
